# Assessment of knowledge, attitudes, and practices among livestock farmers regarding ticks and tick-borne diseases in Bangladesh: A cross-sectional study

**DOI:** 10.1016/j.parepi.2026.e00549

**Published:** 2026-09-30

**Authors:** Md. Hasan Ali, Hemayet Hossain, Niaz Mahmood Tanoy, Md. Tanvir Hasan Bhuiyan, Nandiny Saha Roy, Md. Zubayer Islam, Md. Saiful Islam, Nitto Roy, Sherin Akter Shimu, Md. Ashraful, Aliful Islam Shykat, Athoy Das, Md. Mahfujur Rahman

**Affiliations:** a Faculty of Veterinary, Animal and Biomedical Sciences, Sylhet Agricultural University, Sylhet 3100, Bangladesh; b Department of Anatomy and Histology, Sylhet Agricultural University, Sylhet 3100, Bangladesh; c Department of Veterinary Science and Animal Husbandry, Teesta University, Rangpur 5404, Bangladesh; d Department of Pharmacology and Toxicology, Sylhet Agricultural University, Sylhet 3100, Bangladesh; e Faculty of Veterinary and Animal Science, Hajee Mohammad Danesh Science and Technology University, Dinajpur 5200, Bangladesh; f Department of Medicine, Sylhet Agricultural University, Sylhet 3100, Bangladesh

**Keywords:** Tick-borne diseases, Preventive behavior, Zoonotic disease, KAP survey, Bangladesh

## Abstract

Ticks and tick-borne diseases (TTBDs) remain a major constraint to livestock productivity and a growing public health concern in tropical settings such as Bangladesh. This study aimed to evaluate the knowledge, attitudes, and practices (KAP) of livestock farmers regarding TTBDs and to identify associated determinants. A cross-sectional survey was conducted from 5 January to 15 February 2026 among 530 randomly selected livestock farmers across eight districts using a structured questionnaire, with data analyzed through descriptive statistics, logistic regression, and Spearman's correlation. Only 27.7% of respondents demonstrated good knowledge, despite a high positive attitude (85.5%) and moderately satisfactory practices (45.3%). In multivariate analysis, higher education emerged as a strong predictor of good knowledge, with graduates significantly more likely to be knowledgeable compared to those without formal education (AOR = 10.3, 95% CI: 3.33–36.4, *p* < 0.001), while larger herd size (>50 animals) was also associated with improved knowledge (AOR = 9.20, 95% CI: 3.15–29.7, *p* < 0.001). Receipt of formal training significantly increased the likelihood of satisfactory practices (AOR = 2.78, 95% CI: 1.70–4.62, *p* < 0.001). Knowledge showed a moderate positive correlation with attitudes (*ρ* = 0.574, *p* < 0.001) and practices (*ρ* = 0.410, *p* < 0.001). Despite generally favorable attitudes, critical gaps in knowledge and implementation persist, particularly regarding zoonotic risks and appropriate control strategies. These findings underscore the need for targeted farmer education, strengthened veterinary extension services, and integrated tick management to improve livestock health and productivity. The findings may also inform future One Health-oriented strategies for the prevention and control of tick-borne diseases in Bangladesh.

## Graphical abstract

A cross-sectional KAP survey among 530 livestock farmers across eight districts of Bangladesh revealed low knowledge (27.7%) despite high positive attitudes (85.5%) and moderate practices (45.3%) toward ticks and tick-borne diseases. Education, herd size, and training significantly influenced knowledge and practices. Positive correlations among KAP domains highlight knowledge as a key driver of behavior. Findings emphasize the need for targeted education, veterinary extension services, and integrated tick control within a One Health framework.

**Figure:**
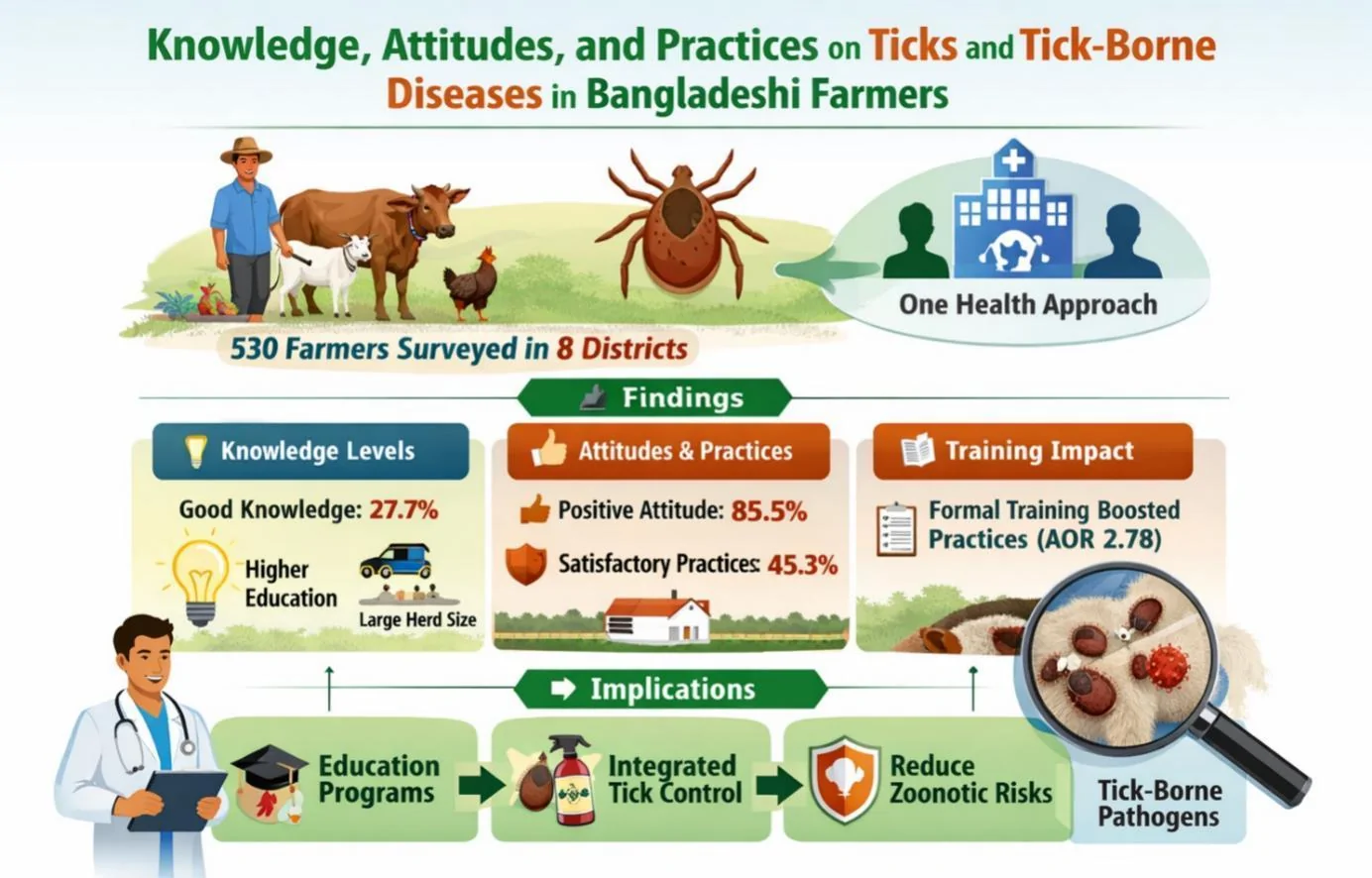

## 1 Introduction

Agriculture is the backbone of the national economy in Bangladesh, and livestock is an integral component of this sector, contributing substantially to food and nutritional security, rural livelihoods, and household income (Ahmed et al., 2015). Livestock resources provide essential products such as meat, milk, and eggs, while supporting farming activities through draft power. This subsector also provides full-time employment to approximately 20% of the population and part-time employment to nearly half of the country's people (Begum et al., 2012). Around 80–85% of rural household's rear cattle as a source of income, along with financial support during periods of economic stress. Despite their economic value, livestock production in Bangladesh is relatively low compared with most of the world (Kamal et al., 2019; Parvez et al., 2014).

Ecto-parasites, such as ticks, mites, lice, and keds, and *endo*-parasites, particularly gastrointestinal worms, are commonly found in livestock. Blood-sucking parasites weaken animals and reduce production performance (Muhammad et al., 2021). Among these ectoparasites, ticks are particularly important because they cause both direct damage to livestock and indirect losses through the transmission of infectious pathogens (Jongejan and Uilenberg, 1994). Ticks are considered one of the most significant threats to livestock. They belong to the order *Acari* and are obligate blood-feeding ectoparasites (Koenen et al., 2012)*.* In addition to causing direct production losses, ticks transmit a wide range of bacterial, protozoal, viral, and rickettsial pathogens, making them one of the most important vectors of veterinary and public health significance (Gong et al., 2025). One of the most widespread species, *Rhipicephalus microplus*, is well known for its role as a vector of pathogens causing bovine babesiosis and anaplasmosis (Uddin et al., 2022). Ticks inflict direct harm on animals by blood loss, skin damage, lesions, secondary infections, and reduced growth performance and milk production (Al-Shammari et al., 2025). Furthermore, ticks are increasingly recognized within the One Health framework because they facilitate pathogen transmission among livestock, wildlife, companion animals, and humans (Eremeeva and Das, 2024).

Tick-borne diseases (TBDs) such as babesiosis and anaplasmosis represent a major concern for livestock production worldwide. Localized control strategies are necessary because tick species diversity and prevalence might differ among regions, host species, and management practices (Bock et al., 2004). Particularly in young or immunocompromised animals, Anaplasmosis, caused by *Anaplasma* species, causes anemia, weight loss, and a decline in milk production (Ierardi, 2025). Anaplasma-specific antibodies were detected in about 70.6% of herds and 42.9% of individual cattle in Bangladesh, according to data from a recent nationwide serological survey. The highest seroprevalence of 74.4% and 45.9%, respectively, was found in the southeast (Zim et al., 2024). These findings indicate the need for better surveillance and control strategies to protect herd health and evidence the extensive distribution of Anaplasma infection among the cattle population in Bangladesh (Zim et al., 2024). Another molecular study found that crossbred cattle had an overall piroplasm infection of 18.7%, with PCR detecting 12.2% *Theileria* and 4.7% *Babesia* (Hossain et al., 2023). *Babesia bigemina* (49.28%) and *Theileria orientalis* (46.01%) were found to be highly prevalent in several regions of Bangladesh in another molecular survey on livestock (Mohanta et al., 2023).

Approximately 80% of the global cattle population is exposed to tick infestation (Strydom et al., 2023). An estimated USD 13.9–30 billion losses occur globally each year, arising from treatment expenses, reduced productivity, and higher susceptibility to other infections carried by ticks and tick-borne diseases (Bose and Siva Kumar, 2025). Integrated tick management, which combines strategic acaricide application, biological control, improved husbandry, and continuous surveillance, is widely recognized as the most sustainable approach for controlling tick infestations and reducing the burden of tick-borne diseases (Abbas et al., 2014). Acaricides remain the primary method of tick control; however, their improper use, inconsistent application, and lack of rotation of active ingredients contribute to the development of acaricide resistance, which reduces control efficacy and increases costs, reducing treatment effectiveness and increasing production costs (Evans et al., 2024).

Livestock farmers play a key role in preventing and controlling tick infestations because they are primarily responsible for implementing routine husbandry and tick control measures (Jamra et al., 2024). However, inadequate awareness regarding housing conditions, feeding practices, hygiene, and biosecurity increases the risk of parasite infestation and disease transmission (Rahman et al., 2013). In veterinary and public health research, the Knowledge, Attitudes, and Practices (KAP) framework is widely used to evaluate community awareness and behavior related to disease prevention and control (Ali et al., 2026; Khan et al., 2024). KAP studies provide a systematic approach for identifying knowledge gaps, misconceptions, and behavioral barriers that influence disease prevention, thereby facilitating the development of targeted educational interventions and evidence-based veterinary extension programmes (Hossain et al., 2026; Launiala, 2009). Research from several countries, including India (Jamra et al., 2024), Bhutan (Namgyal et al., 2021), Pakistan (Hussain et al., 2021), Nigeria (Malgwi et al., 2025), Tunisia (Khamassi Khbou et al., 2025) has reported on farmers' knowledge, attitudes, and practices regarding ticks and tick-borne diseases.

The success of tick control programs largely relies on developing a good understanding of farmers' knowledge about ticks and TBDs, their perceptions of the effectiveness of the proposed control methods, and the socio-cultural context in which such programs are to be implemented. By improving farmer awareness, the adoption of integrated tick management strategies can promote safer use of acaricides and improve animal health outcomes.

Although a previous study has investigated knowledge, attitudes, and practices regarding ticks and tick-borne diseases in Bangladesh, comprehensive, large-scale, multi-district KAP assessments among livestock farmers remain limited. The purpose of this study was to evaluate the knowledge, attitudes, and practices toward tick-borne diseases among livestock farmers in Bangladesh. The findings may contribute to the development of targeted intervention strategies, including improved farmer education, strengthened veterinary extension services, and effective tick control and herd management programs in Bangladesh.

## 2 Method and materials

### 2.1 Ethical statement

This study was conducted following approval from the Institutional Ethics Committee of Teesta University (Approval No. *TU/IEC/2025/004*).

### 2.2 Study area

The KAP survey on ticks and tick-borne diseases was conducted from 5 January to 15 February 2026 across eight districts of Bangladesh: Natore, Cumilla, Sylhet, Brahmanbaria, Gazipur, Narail, Dinajpur, and Chattogram (Fig. 1). These districts were strategically selected to capture a diverse demographic of livestock farmers and varied ecological conditions, including tea gardens, hilly shrublands, and forested landscapes, which provide favorable environments for tick survival and proliferation. Participants were recruited in each district using random sampling. The study encompassed diverse livestock species, including cattle, buffalo, sheep, goats, and mixed breeds, across urban, semi-urban, and rural settings.

**Fig. 1:**
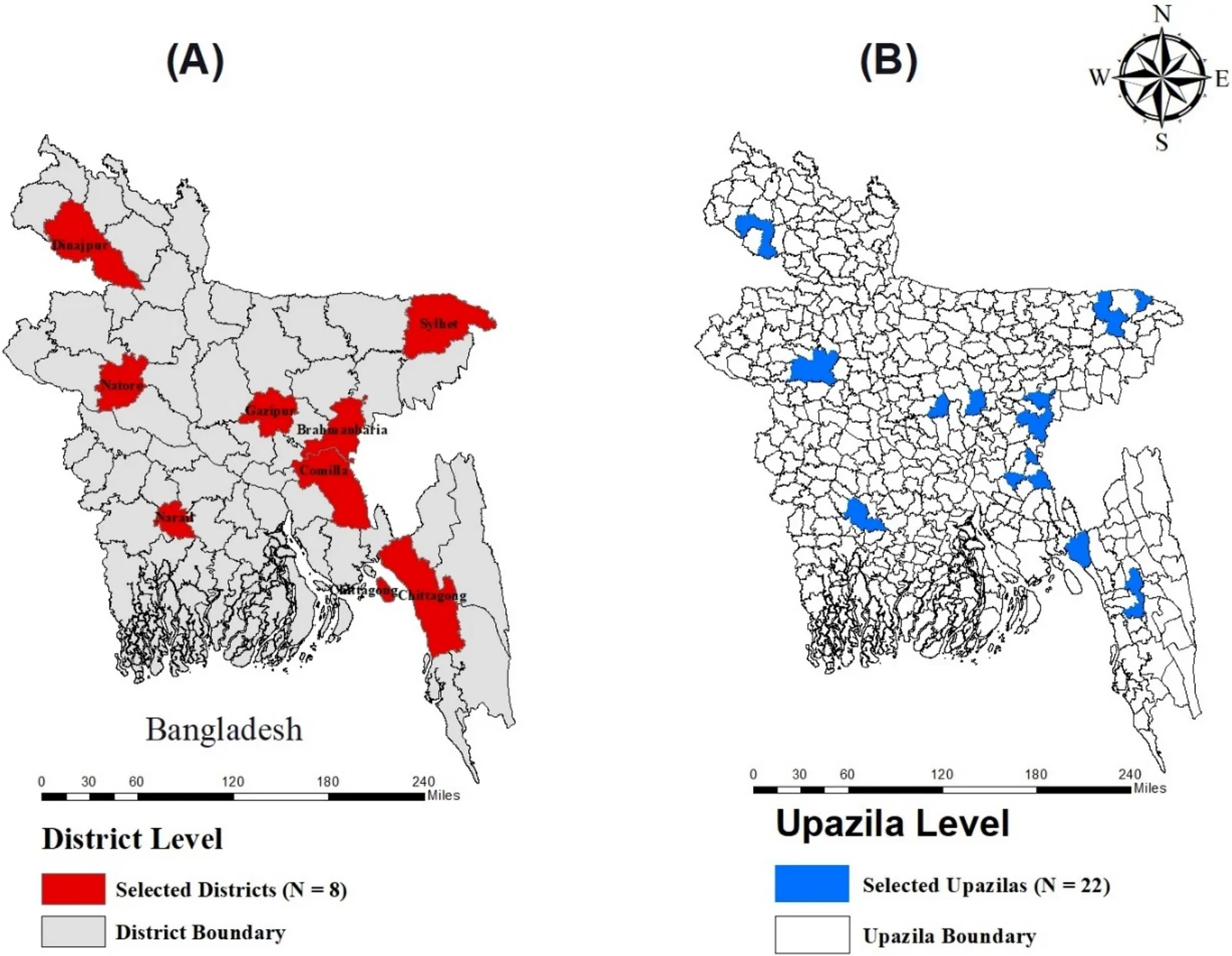
Geographic location of the study area in Bangladesh, illustrating (A) the district-level distribution and (B) the upazila-level distribution of the study sites. The map was prepared using ArcMap version 10.8.

### 2.3 Sample size calculation

The required number of samples was estimated with the following formula (Naing et al., 2022).

$$n=\frac{Z^{2}\times \text{Pexp}\times \left(1-\text{Pexp}\right)}{d^{2}}$$

Where, n = Desired sample size;

Z = 1.96 for 95% confidence interval;

P_exp_ = 0.5, Expected proportion of farmers estimated to have certain knowledge, attitudes, and practices.

d = 0.05, Desired absolute precision (5%).

Therefore, the minimum required sample size (n) was determined to be 384. Nevertheless, data were collected from 530 participants, exceeding the minimum requirement, to enhance the statistical power and reliability of the results.

### 2.4 Study design

This cross-sectional study involved a total of 530 livestock farmers, selected based on predefined eligibility criteria. At least 60 farmers were randomly selected from each study district to ensure adequate representation across the study areas. Participants included individuals actively engaged in animal husbandry, those involved in related activities, and those who had received formal training in livestock management. Eligibility criteria further considered livestock housing, at least 1 year of farming experience, ownership of a minimum of two animals, and willingness to participate. Farmers who did not meet these criteria or declined participation were excluded. Before data collection, participants were informed about the study objectives, methodology, potential risks, and anticipated benefits, and verbal informed consent was obtained. During the survey, misconceptions regarding ticks and tick-borne diseases were addressed using scientifically accepted information. A pilot study involving 30 farmers was conducted to refine the questionnaire, and feedback was incorporated into the final instrument. Data from the pilot were excluded from the main analysis to maintain consistency and accuracy.

### 2.5 Questionnaire

A structured questionnaire was developed to collect comprehensive data on multiple aspects of ticks and tick-borne diseases. The questionnaire was designed based on an extensive literature review and input from experienced epidemiological investigators, and it was self-administered during face-to-face interviews with animal owners. It comprised 44 well-structured questions divided into four sections: socio-demographic information, knowledge, attitudes, and preventive practices related to TTBDs. The socio-demographic section included 10 questions covering participants' age, gender, district/upazila, educational level, place of residence, farming experience, herd size, formal training, types of animals reared, and grazing management. The knowledge section comprised 12 questions evaluating participants' awareness of TTBDs, with response options of “Yes” for correct answers, “No” for incorrect answers, and “Don't know” for uncertainty. The attitudes section included 11 questions, each rated on a five-point Likert scale (Strongly Agree to Strongly Disagree) to assess participants' perceptions regarding TTBDs. The preventive practices section consisted of 11 questions, also measured on a five-point Likert scale (Always to Never), to evaluate the implementation of TTBD control measures among farmers.

### 2.6 Data collection

Data were collected using the KoboToolbox platform (https://kf.kobotoolbox.org) (Kobo Collect, Version 2.025.10) on Android tablets and smartphones. Data collection was conducted by a team of trained research assistants, including veterinary and public health graduates with prior experience in community-based surveys, under the supervision of the lead researcher. Before field deployment, all enumerators received comprehensive training on the study objectives, data collection procedures, ethical considerations, and confidentiality protocols to ensure standardized and reliable data collection. The electronic questionnaire was programmed with skip logic, mandatory fields, and validation checks to minimize entry errors and improve data quality. During each interview, one enumerator administered the questionnaire while another supervised and verified the responses, ensuring completeness and accuracy. All collected data were securely stored on the KoboToolbox server with restricted access, thereby maintaining participant privacy and confidentiality throughout the study.

### 2.7 Assessment of internal consistency

The total Cronbach's alpha for this questionnaire was calculated to be 0.75, which signifies acceptable reliability. An acceptable reliability value is greater than 0.60, and an outstanding reliability value is greater than 0.80, indicating high internal consistency. The internal consistency and reliability of the questionnaire were determined with Cronbach's alpha, which supports its use in this research (Ursachi et al., 2015).

### 2.8 KAP scores

Participants' knowledge, attitudes, and practices (KAP) were quantified using structured scoring systems. Knowledge was assessed using a binary scoring approach, where responses of “Yes” were assigned a score of 1, while “No” or “Don't know” were scored as 0, reflecting the level of respondents' understanding. Attitudes were measured using a five-point Likert scale ranging from 5 (“Strongly Agree”) to 1 (“Strongly Disagree”). Preventive practices were evaluated using a five-point frequency scale, with scores ranging from 1 (“Never”) to 5 (“Always”). The maximum possible scores for the knowledge, attitude, and practice domains were 12, 55, and 55, respectively. Domain-specific scores were calculated by summing item-level responses and subsequently converting them into percentage values. Based on these percentages, respondents were categorized as having “Good,” “Positive,” and “Satisfactory” levels of knowledge, attitudes, and practices, respectively, if their scores were ≥ 65%, while scores <65% were classified as “Poor,” “Negative,” and “Unsatisfactory.” Multiple-choice questions were excluded from the overall scoring to avoid potential bias in the quantitative assessment (Hossain et al., 2025).

### 2.9 Statistical analysis

Only completed questionnaires were included in the final analysis. Statistical analyses were performed using R (version 4.3.4). Reproducible descriptive tables were generated using the gtsummary package (Sjoberg et al., 2021). Categorical variables were summarized using frequencies and percentages. Univariate and multivariate logistic regression analyses were conducted to identify factors associated with the levels of knowledge, attitudes, and practices (KAP) regarding ticks and tick-borne diseases. Statistical significance was determined at a *p*-value <0.05 with a 95% confidence interval. Adjusted odds ratios (AORs) were calculated to estimate the strength of association between significant factors and KAP outcomes. In addition, Spearman's rank correlation coefficient was applied to examine the relationships among knowledge, attitudes, and practices.

## 3 Results

### 3.1 Sociodemographic characteristics of the respondents

A total of 530 participants were included in the study. Respondents were recruited from multiple districts of Bangladesh, including Brahmanbaria (13.4%), Gazipur (13.2%), Narail (11.5%), Natore (13.2%), Sylhet (12.3%), Dinajpur (13.8%), Cumilla (11.3%), and Chattogram (11.3%), indicating broad geographical representation (Table 1). In terms of age distribution, the largest proportion of participants belonged to the 30–40 years age group (32.1%), followed by ≥50 years (27.5%) and 41–50 years (27.0%), while 13.4% were below 30 years of age. The majority of respondents were male (57.5%), whereas females constituted 42.5% of the study population. Regarding educational attainment, 26.6% of participants had no formal education. Approximately one-quarter (24.9%) had completed primary education, and 29.2% had completed secondary education. A smaller proportion had higher secondary education (15.1%) or graduate-level education and above (4.2%). Most respondents resided in rural areas (63.0%), followed by semi-urban (29.4%) and urban settings (7.5%). In terms of farming experience, 33.2% had 5–10 years of experience, 24.9% had less than 5 years, 21.9% had 11–20 years, and 20.0% reported more than 20 years of farming experience. Cattle farming was the predominant livestock practice (58.9%), followed by mixed livestock rearing (27.4%) and goat/sheep farming (12.3%), while buffalo farming was comparatively rare (1.5%). With respect to herd/flock size, 45.7% of respondents maintained fewer than five animals, 46.0% reared 5–20 animals, 4.2% had 21–50 animals, and 4.2% reported more than 50 animals. Notably, only 24.3% of participants reported having received formal training on animal health or disease prevention, whereas the majority (75.7%) had not received any formal training. Slightly more than half of the respondents (51.3%) practiced semi-intensive or free-grazing management systems, while 48.7% did not.

**Table 1:** Sociodemographic characteristics of respondents (*N* = 530).

Characteristic	Frequency (%)
District/Upazila	
Brahmanbaria	71 (13.4%)
Chattogram	60 (11.3%)
Cumilla	60 (11.3%)
Dinajpur	73 (13.8%)
Gazipur	70 (13.2%)
Narail	61 (11.5%)
Natore	70 (13.2%)
Sylhet	65 (12.3%)
Age (years)	
<30	71 (13.4%)
30–40	170 (32.1%)
41–50	143 (27.0%)
50+	146 (27.5%)
Gender	
Male	305 (57.5%)
Female	225 (42.5%)
Educational level	
No formal education	141 (26.6%)
Primary	132 (24.9%)
Secondary	155 (29.2%)
Higher secondary	80 (15.1%)
Graduate or above	22 (4.2%)
Place of residence	
Rural	334 (63.0%)
Semi urban	156 (29.4%)
Urban	40 (7.5%)
Farming experience	
<5	132 (24.9%)
5–10	176 (33.2%)
11–20	116 (21.9%)
>20	106 (20.0%)
Type of livestock reared	
Goat/Sheep	65 (12.3%)
Cattle	312 (58.9%)
Buffalo	8 (1.5%)
Mixed	145 (27.4%)
Herd/flock size	
<5	242 (45.7%)
5–20	244 (46.0%)
21–50	22 (4.2%)
>50	22 (4.2%)
Have you ever received formal training on animal health or disease prevention?	
No	401 (75.7%)
Yes	129 (24.3%)
Do you practice semi-intensive or free grazing management for your animals?	
Yes	272 (51.3%)
No	258 (48.7%)

### 3.2 Level of knowledge, attitude, and practice regarding TTBDs

Among the 530 livestock farmers surveyed, less than one-third (27.7%, *n* = 147) demonstrated good knowledge of ticks and tick-borne diseases, whereas the majority (72.3%, *n* = 383) had poor knowledge (Table 2). Attitudinal assessment revealed that most participants (85.5%, *n* = 453) held a positive attitude, whereas 14.5% (77) exhibited a negative attitude. In terms of management practices, 45.3% (240) followed satisfactory practices, whereas 54.7% demonstrated unsatisfactory practices (Table 2).

**Table 2:** Level of knowledge, attitude, and practice regarding Tick and Tick-borne disease.

Characteristic	*N* = 530^*1*^
Knowledge level	
Good	147 (27.7%)
Poor	383 (72.3%)
Attitude level	
Positive	453 (85.5%)
Negative	77 (14.5%)
Practice level	
Satisfactory	240 (45.3%)
Unsatisfactory	290 (54.7%)

#### 3.2.1 Distribution of participants' knowledge

A total of 530 livestock farmers were surveyed to assess their knowledge of ticks and tick-borne diseases (TTBDs) (Supplementary Table 1). Most participants (78.5%, *n* = 416) correctly identified ticks as external blood-feeding parasites, and 55.5% (*n* = 294) recognized their role in transmitting infectious diseases to livestock (Fig. 2). While 65.1% (*n* = 345) acknowledged that TTBDs reduce productivity, including milk yield, meat production, and growth, only 42.5% (*n* = 225) appreciated the potential severity of untreated infections, and awareness of zoonotic transmission was limited (32.1%, *n* = 170). Knowledge of environmental factors and management practices was moderate: 39.8% (*n* = 211) identified warm, humid conditions as favorable for ticks, 41.3% (*n* = 219) recognized seasonal variation, and 48.9% (*n* = 259) perceived free-grazing animals as more susceptible. Regarding control, 68.1% (*n* = 361) reported that proper measures can prevent TTBDs, yet awareness of acaricide resistance due to misuse was low (26.8%, *n* = 142). Early diagnosis and treatment were correctly associated with improved recovery by 65.8% (*n* = 349), whereas knowledge of specific infections such as anaplasmosis, babesiosis, and theileriosis remained limited (23.4%, *n* = 124).

**Fig. 2:**
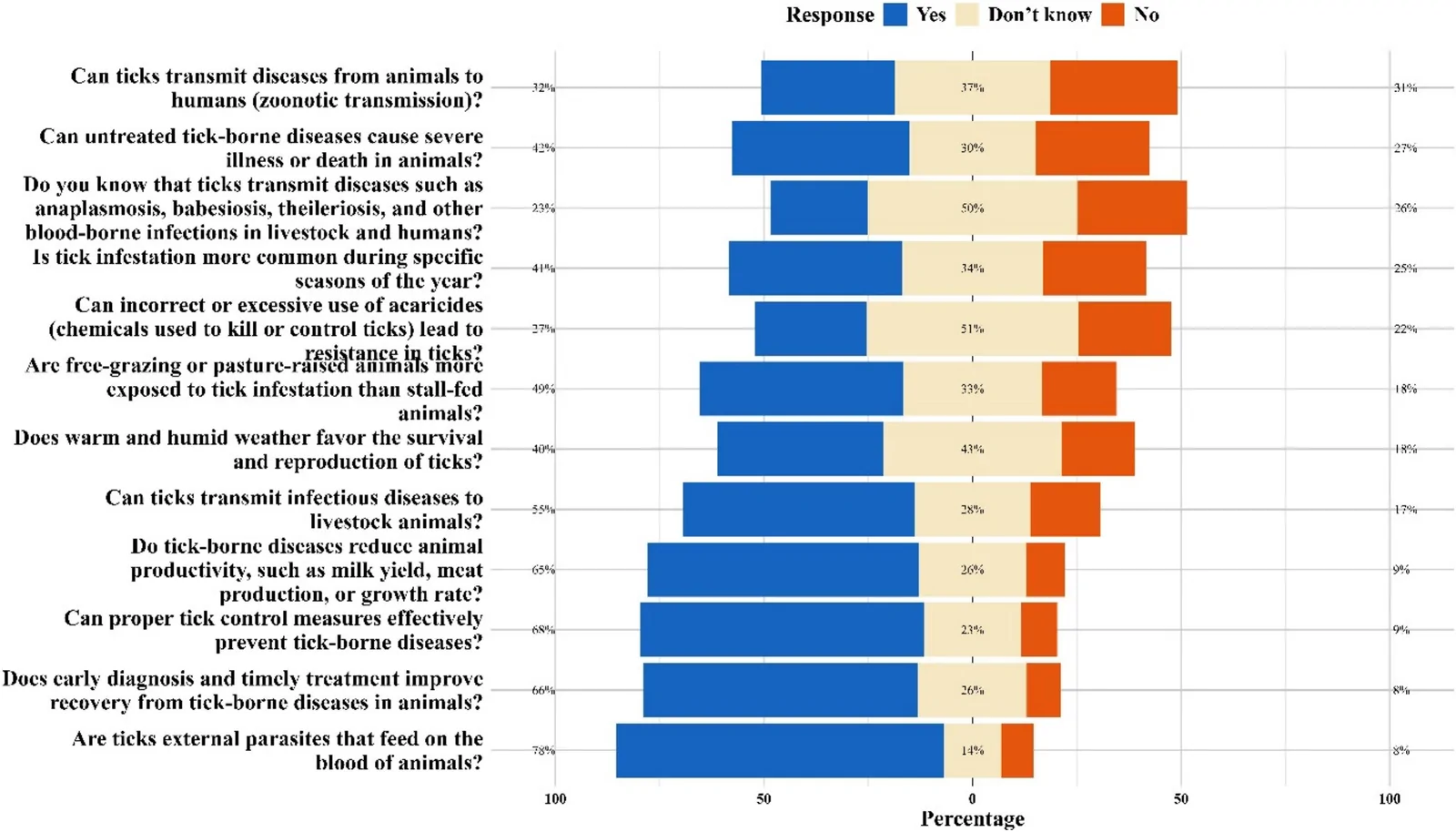
Distribution of participants' knowledge regarding ticks and tick-borne diseases.

#### 3.2.2 Distribution of participants' attitudes

Among the 530 livestock farmers surveyed, attitudes toward ticks and tick-borne diseases (TTBDs) demonstrated a strong recognition of their significance, yet also revealed areas of uncertainty or disagreement (Supplementary Table 2). Most participants acknowledged tick infestation as a serious livestock health problem (strongly agree: 25.7%, agree: 45.7%), although 22.8% were neutral and 5.9% disagreed (Fig. 3). Controlling ticks as a regular and essential part of farm management was supported by 63.2% (strongly agree: 21.7%, agree: 41.5%), while nearly one-third (31.5%) were neutral, and a small proportion (4.3%) disagreed. Similarly, the economic impact of TTBDs was recognized by 61.7% (strongly agree: 18.1%, agree: 43.6%), yet 30.6% remained neutral, and 7.8% disagreed. Preventive measures were generally favored, with 62.5% endorsing prevention over treatment, though 31.7% were neutral and 5.9% disagreed. Farmers' personal role in controlling ticks was acknowledged by 69.8% (strongly agree: 28.9%, agree: 40.9%), yet 26.8% were neutral, and 3.4% disagreed. Regular use of acaricides was considered necessary by 43.4% (strongly agree: 12.1%, agree: 31.3%), but 54.5% were neutral or disagreed, indicating hesitancy or knowledge gaps. Veterinary consultation was deemed important by 49.5% (strongly agree: 15.3%, agree: 34.2%), while 35.5% were neutral and 15.1% disagreed. Poor farm hygiene was widely recognized as increasing tick risk (strongly agree: 51.3%, agree: 26.2%), with 17.5% neutral and 5.0% disagreeing. Reporting severe infestations to veterinarians was considered important by 50.3% (strongly agree: 10.9%, agree: 39.4%), though 39.6% were neutral and 10% disagreed. Training farmers on tick control practices was supported by over 60% (strongly agree: 13.6–14.7%, agree: 47.4–47.7%), with approximately one-third neutral and a small minority opposing..

**Fig. 3:**
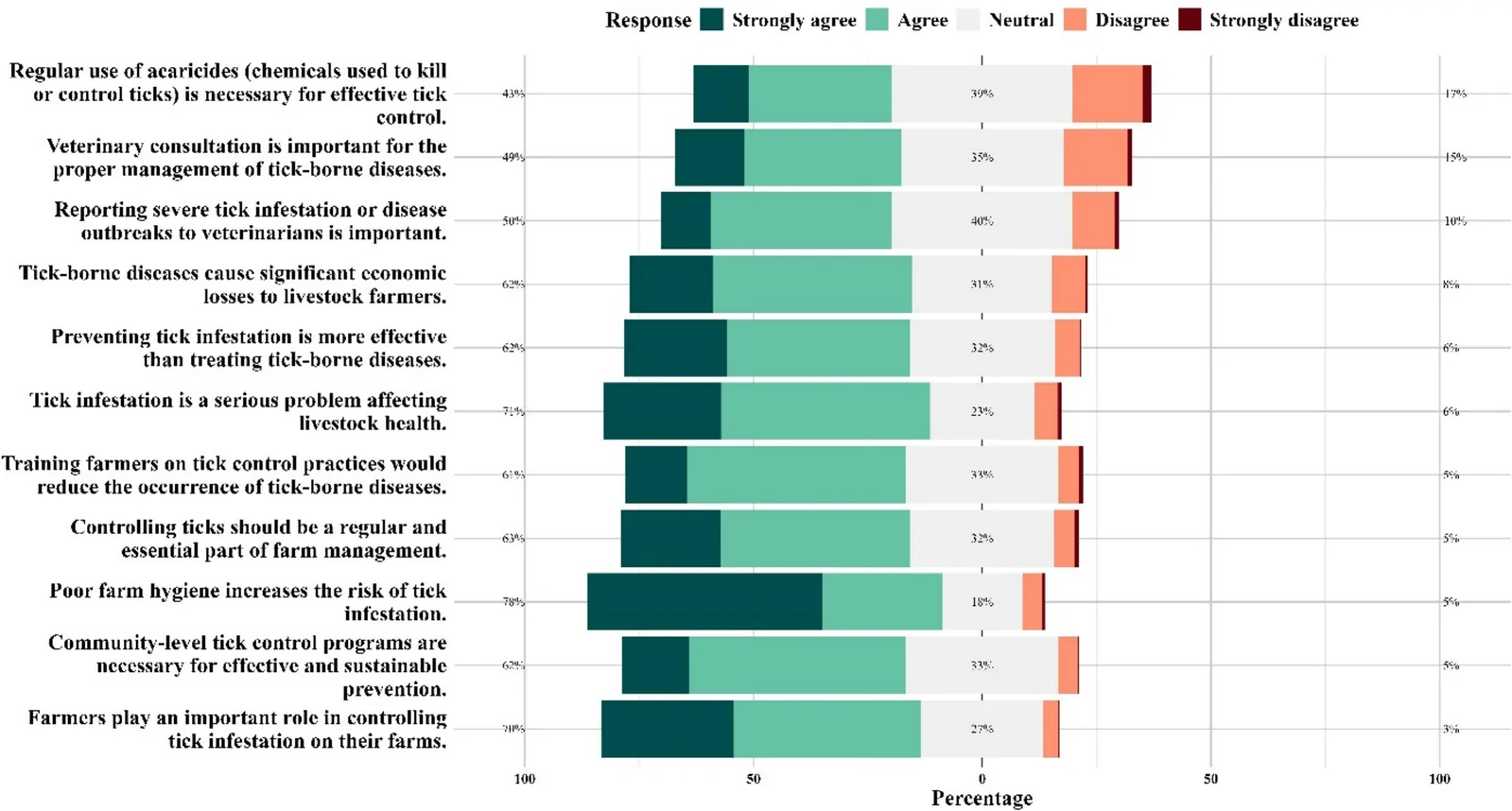
Distribution of participants' attitudes regarding ticks and tick-borne diseases (*N* = 530).

#### 3.2.3 Distribution of participants' practices

Participants' practices related to tick and tick-borne disease control were generally inconsistent (Supplementary Table 3). Only 30.4% of respondents reported always checking animals for ticks (*n* = 161), whereas a substantial proportion performed this practice irregularly. Manual removal of visible ticks was more common, with 41.1% always adopting this measure (*n* = 218). In contrast, consistent use of acaricides was limited; only 15.5% always applied acaricides (*n* = 82), while 24.3% reported never using them. Adherence to recommended veterinary or manufacturer instructions for acaricide dosage was particularly low, with just 13.2% always following guidelines (*n* = 60). Farm hygiene practices were comparatively better, as nearly half of the participants always cleaned and maintained animal housing to reduce tick habitats (48.5%, *n* = 257). Veterinary consultation remained limited, with only 7.7% consistently seeking professional advice for animals showing signs of tick-borne diseases. Biosecurity and environmental management practices were poorly implemented, including isolation of infested animals (11.5%), treatment of newly purchased animals before herd integration (14.9%), and rotation of grazing areas to reduce tick exposure (10.4%). Record-keeping was notably inadequate, with 44.0% never maintaining records of tick infestation or treatments (*n* = 233), and only 5.7% consistently using personal protective measures when handling tick-infested animals (Fig. 4).

**Fig. 4:**
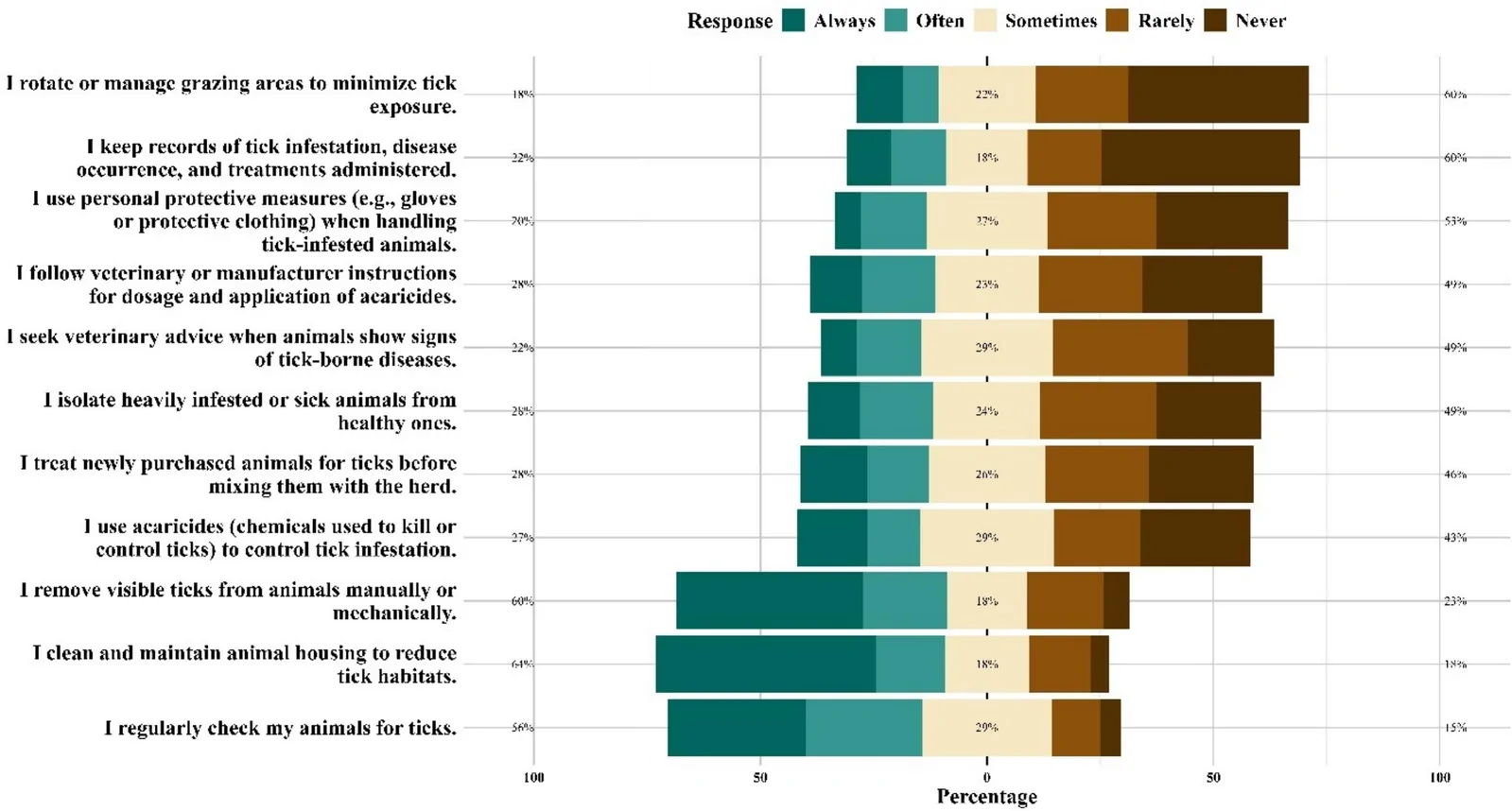
Distribution of participants' practices regarding ticks and tick-borne diseases (*N* = 530).

### 3.3 Factors associated with the level of knowledge measures

Associations between socio-demographic characteristics and respondents' knowledge regarding ticks and tick-borne diseases were evaluated using univariate and multivariate logistic regression analyses (Table 3). In the univariate analysis, gender, education level, farming experience, herd size, and receipt of formal training on animal health or disease prevention were significantly associated with knowledge level (*p* < 0.05). However, after adjustment in the multivariate model, gender, education level, farming experience, and herd size remained significant predictors of good knowledge. Female respondents were significantly more likely to have good knowledge compared with males (AOR = 2.47, 95% CI: 1.54–4.01, *p* < 0.001). Education showed a strong positive association with knowledge, with respondents having a graduate degree or higher demonstrating substantially greater odds of good knowledge than those with no formal education (AOR = 10.3, 95% CI: 3.33–36.4, *p* < 0.001). Farming experience was also significantly associated with knowledge; respondents with 11–20 years (AOR = 2.31, 95% CI: 1.21–4.52, *p* = 0.012) and 5–10 years of experience (AOR = 2.13, 95% CI: 1.11–3.80, *p* = 0.024) had higher odds of good knowledge compared with those having less than 5 years of experience. Herd size was another important determinant, as farmers managing more than 50 animals had markedly higher odds of good knowledge than those with fewer than five animals (AOR = 9.20, 95% CI: 3.15–29.7, *p* < 0.001), whereas herd sizes of 21–50 animals were not statistically significant after adjustment (*p* = 0.058).

**Table 3:** Factors associated with the level of knowledge regarding ticks and tick-borne diseases.

	Knowledge level		Univariate			Multivariate		
Characteristic	Good *N* = 147	Poor *N* = 383	COR	95% CI	*p*-value	AOR	95% CI	p-value
Age (years)								
<30	23 (16%)	48 (13%)	*Ref*	–		–	–	
30–40	38 (26%)	132 (34%)	0.60	0.33, 1.12	0.10	0.76	0.38, 1.54	0.4
41–50	34 (23%)	109 (28%)	0.65	0.35, 1.23	0.2	0.76	0.37, 1.58	0.5
50+	52 (35%)	94 (25%)	1.15	0.64, 2.13	0.6	1.92	0.95, 3.96	0.072
Gender								
Male	71 (48%)	234 (61%)	*Ref*	–		–	–	
Female	76 (52%)	149 (39%)	1.68	1.15, 2.47	0.008	2.47	1.54, 4.01	<0.001
Educational level								
No formal education	30 (20%)	111 (29%)	*Ref*	–		–	–	
Primary	31 (21%)	101 (26%)	1.14	0.64, 2.01	0.7	1.25	0.66, 2.37	0.5
Secondary	41 (28%)	114 (30%)	1.33	0.78, 2.29	0.3	1.53	0.84, 2.81	0.2
Higher secondary	28 (19%)	52 (14%)	1.99	1.08, 3.68	0.027	1.70	0.82, 3.52	0.2
Graduate or above	17 (12%)	5 (1.3%)	12.6	4.57, 40.9	<0.001	10.3	3.33, 36.4	<0.001
Place of residence								
Rural	84 (57%)	250 (65%)	*Ref*	–		–	–	
Semi urban	51 (35%)	105 (27%)	1.45	0.95, 2.19	0.082	1.12	0.68, 1.84	0.6
Urban	12 (8.2%)	28 (7.3%)	1.28	0.60, 2.57	0.5	1.09	0.46, 2.47	0.8
Farming experience								
<5	24 (16%)	108 (28%)	*Ref*	–		–	–	
>20	31 (21%)	75 (20%)	1.86	1.01, 3.45	0.046	1.40	0.69, 2.87	0.4
11–20	38 (26%)	78 (20%)	2.19	1.22, 3.99	0.009	2.31	1.21, 4.52	0.012
5–10	54 (37%)	122 (32%)	1.99	1.16, 3.49	0.013	2.03	1.11, 3.80	0.024
Type of livestock reared								
Goat/Sheep	16 (11%)	49 (13%)	*Ref*	–		–	–	
Cattle	80 (54%)	232 (61%)	1.06	0.58, 2.01	0.9	1.45	0.70, 3.15	0.3
Buffalo	4 (2.7%)	4 (1.0%)	3.06	0.66, 14.4	0.14	4.16	0.75, 23.4	0.10
Mixed	47 (32%)	98 (26%)	1.47	0.77, 2.91	0.3	1.60	0.76, 3.51	0.2
Herd/flock size								
<5	49 (33%)	193 (50%)	*Ref*	–		–	–	
>50	16 (11%)	6 (1.6%)	10.5	4.09, 30.6	<0.001	9.20	3.15, 29.7	<0.001
21–50	10 (6.8%)	12 (3.1%)	3.28	1.31, 8.06	0.009	2.68	0.95, 7.42	0.058
5–20	72 (49%)	172 (45%)	1.65	1.09, 2.51	0.019	1.46	0.90, 2.39	0.12
Have you ever received formal training on animal health or disease prevention?								
No	98 (67%)	303 (79%)	*Ref*	–		–	–	
Yes	49 (33%)	80 (21%)	1.89	1.24, 2.88	0.003	1.36	0.80, 2.28	0.3
Do you practice semi-intensive or free grazing management for your animals?								
No	63 (43%)	195 (51%)	*Ref*	–		–	–	
Yes	84 (57%)	188 (49%)	1.38	0.94, 2.03	0.10	1.40	0.88, 2.23	0.2

Abbreviations: AOR, adjusted odds ratio; CI, confidence interval; COR, crude odds ratio. Reference categories: Age: <30 years; Gender: Male; Educational level: No formal education; Place of residence: Rural; Farming experience: <5 years; Type of livestock reared: Goat/Sheep; Herd/flock size: <5 animals; Formal training on animal health or disease prevention: No; Semi-intensive or free grazing management: No. *P* < 0.05 was considered statistically significant.

### 3.4 Factors associated with the level of attitude measures

Associations between socio-demographic characteristics and respondents' attitudes toward ticks and tick-borne diseases were examined using univariate and multivariate logistic regression analyses (Table 4). In the univariate analysis, none of the assessed variables showed a statistically significant association with attitude level (*p* > 0.05), although urban residence showed a marginal association (*p* = 0.054). Similarly, the multivariate logistic regression model did not identify any significant predictors of attitude toward ticks and tick-borne diseases. For instance, respondents with higher secondary education had higher odds of positive attitudes compared with those with no formal education (AOR = 2.58, 95% CI: 0.64–10.44), while those managing herds of more than 50 animals showed lower odds of positive attitudes compared with respondents managing fewer than five animals (AOR = 2.21, 95% CI: 0.38–42.0); however, these associations were not statistically significant (*p* > 0.05).

**Table 4:** Factors associated with the level of attitude regarding ticks and tick-borne diseases.

	Attitude level		Univariate			Multivariate		
Characteristic	Negative *N* = 77	Positive *N* = 453	COR	95% CI	p-value	AOR	95% CI	p-value
Age (years)								
<30	8 (10%)	63 (14%)	*Ref*	–		–	–	
30–40	28 (36%)	142 (31%)	0.64	0.26, 1.43	0.3	0.58	0.22, 1.35	0.2
41–50	23 (30%)	120 (26%)	0.66	0.26, 1.51	0.3	0.63	0.24, 1.52	0.3
50+	18 (23%)	128 (28%)	0.90	0.35, 2.13	0.8	0.87	0.32, 2.23	0.8
Gender								
Male	44 (57%)	261 (58%)	*Ref*	–		–	–	
Female	33 (43%)	192 (42%)	0.98	0.60, 1.61	>0.9	1.11	0.63, 1.97	0.7
Educational level								
No formal education	22 (29%)	119 (26%)	*Ref*	–		–	–	
Primary	14 (18%)	118 (26%)	1.56	0.77, 3.26	0.2	1.36	0.64, 2.94	0.4
Secondary	33 (43%)	122 (27%)	0.68	0.37, 1.23	0.2	0.62	0.32, 1.17	0.15
Higher secondary	7 (9.1%)	73 (16%)	1.93	0.82, 5.08	0.2	1.58	0.62, 4.44	0.4
Graduate or above	1 (1.3%)	21 (4.6%)	3.88	0.75, 71.4	0.2	2.53	0.44, 48.0	0.4
Place of residence								
Rural	52 (68%)	282 (62%)	*Ref*	–		–	–	
Semi urban	24 (31%)	132 (29%)	1.01	0.61, 1.74	>0.9	0.85	0.48, 1.55	0.6
Urban	1 (1.3%)	39 (8.6%)	7.19	1.51, 129	0.054	6.29	1.23, 115	0.079
Farming experience								
<5	19 (25%)	113 (25%)	*Ref*	–		–	–	
>20	18 (23%)	88 (19%)	0.82	0.41, 1.67	0.6	0.72	0.33, 1.54	0.4
11–20	20 (26%)	96 (21%)	0.81	0.40, 1.60	0.5	0.87	0.42, 1.79	0.7
5–10	20 (26%)	156 (34%)	1.31	0.67, 2.58	0.4	1.14	0.56, 2.31	0.7
Type of livestock reared								
Goat/Sheep	14 (18%)	51 (11%)	*Ref*	–		–	–	
Cattle	44 (57%)	268 (59%)	1.67	0.83, 3.21	0.13	1.20	0.55, 2.50	0.6
Buffalo	2 (2.6%)	6 (1.3%)	0.82	0.17, 6.04	0.8	0.56	0.08, 4.93	0.6
Mixed	17 (22%)	128 (28%)	2.07	0.94, 4.50	0.067	1.77	0.77, 4.02	0.2
Herd/flock size								
<5	35 (45%)	207 (46%)	*Ref*	–		–	–	
>50	1 (1.3%)	21 (4.6%)	3.55	0.71, 64.7	0.2	2.21	0.38, 42.0	0.5
21–50	1 (1.3%)	21 (4.6%)	3.55	0.71, 64.7	0.2	3.88	0.70, 73.0	0.2
5–20	40 (52%)	204 (45%)	0.86	0.52, 1.41	0.6	0.87	0.50, 1.51	0.6
Have you ever received formal training on animal health or disease prevention?								
No	65 (84%)	336 (74%)	*Ref*	–		–	–	
Yes	12 (16%)	117 (26%)	1.89	1.02, 3.78	0.056	1.69	0.84, 3.70	0.2
Do you practice semi-intensive or free grazing management for your animals?								
No	32 (42%)	226 (50%)	*Ref*	–		–	–	
Yes	45 (58%)	227 (50%)	0.71	0.44, 1.16	0.2	0.76	0.43, 1.31	0.3

Abbreviations: AOR, adjusted odds ratio; CI, confidence interval; COR, crude odds ratio. Reference categories: Age: <30 years; Gender: Male; Educational level: No formal education; Place of residence: Rural; Farming experience: <5 years; Type of livestock reared: Goat/Sheep; Herd/flock size: <5 animals; Formal training on animal health or disease prevention: No; Semi-intensive or free grazing management: No. *P* < 0.05 was considered statistically significant.

### 3.5 Factors associated with the level of practice measures

Associations between respondents' socio-demographic characteristics and their practices regarding tick and tick-borne disease prevention were assessed using univariate and multivariate logistic regression analyses (Table 5). In the univariate analysis, several variables, including education level, place of residence, herd size, type of livestock reared, and receipt of formal training on animal health or disease prevention, showed significant associations with practice level (*p* < 0.05). However, after adjustment in the multivariate model, only selected factors remained significantly associated with satisfactory practice levels. Respondents aged 30–40 years demonstrated higher odds of satisfactory practices compared with those aged <30 years (AOR = 1.96, 95% CI: 1.03–3.83, *p* = 0.044), while those aged ≥50 years also showed significantly greater odds of satisfactory practices (AOR = 2.44, 95% CI: 1.23–4.93, *p* = 0.012). Education level was strongly associated with practice, with respondents having a graduate degree or above being substantially more likely to demonstrate satisfactory practices than those with no formal education (AOR = 7.94, 95% CI: 2.77–46.5, *p* = 0.001). Place of residence was another significant determinant, as respondents living in urban areas had higher odds of satisfactory practices compared with those living in rural areas (AOR = 4.84, 95% CI: 2.06–12.5, *p* < 0.001). Herd size was also significantly associated with practice level; respondents managing herds of 21–50 animals (AOR = 3.79, 95% CI: 1.28–12.4, *p* = 0.020) and those with 5–20 animals (AOR = 1.85, 95% CI: 1.20–2.89, *p* = 0.006) showed significantly higher odds of satisfactory practices compared with those managing fewer than five animals. Furthermore, respondents who had received formal training on animal health or disease prevention were significantly more likely to report satisfactory practices than those who had not received such training (AOR = 2.78, 95% CI: 1.70–4.62, *p* < 0.001).

**Table 5:** Factors associated with the level of practice regarding ticks and tick-borne diseases.

	Practice level		Univariate			Multivariate		
Characteristic	Satisfactory *N* = 240	Unsatisfactory *N* = 290	COR	95% CI	p-value	AOR	95% CI	p-value
Age (years)								
<30	28 (12%)	43 (15%)	*Ref*	–		–	–	
30–40	79 (33%)	91 (31%)	1.33	0.76, 2.36	0.3	1.96	1.03, 3.83	0.044
41–50	59 (25%)	84 (29%)	1.08	0.61, 1.94	0.8	1.46	0.74, 2.89	0.3
50+	74 (31%)	72 (25%)	1.58	0.89, 2.83	0.12	2.44	1.23, 4.93	0.012
Gender								
Male	144 (60%)	161 (56%)	*Ref*	–		–	–	
Female	96 (40%)	129 (44%)	0.83	0.59, 1.18	0.3	1.09	0.71, 1.68	0.7
Educational level								
No formal education	50 (21%)	91 (31%)	*Ref*	–		–	–	
Primary	51 (21%)	81 (28%)	1.15	0.70, 1.88	0.6	0.85	0.48, 1.49	0.6
Secondary	66 (28%)	89 (31%)	1.35	0.85, 2.16	0.2	1.08	0.63, 1.86	0.8
Higher secondary	54 (23%)	26 (9.0%)	3.78	2.13, 6.84	<0.001	3.55	1.80, 7.13	<0.001
Graduate or above	19 (7.9%)	3 (1.0%)	11.5	3.70, 50.8	<0.001	9.74	2.77, 46.5	0.001
Place of residence								
Rural	131 (55%)	203 (70%)	*Ref*	–		–	–	
Semi urban	77 (32%)	79 (27%)	1.51	1.03, 2.22	0.035	0.87	0.54, 1.37	0.5
Urban	32 (13%)	8 (2.8%)	6.20	2.90, 14.8	<0.001	4.84	2.06, 12.5	<0.001
Farming experience								
<5	55 (23%)	77 (27%)	*Ref*	–		–	–	
>20	49 (20%)	57 (20%)	1.20	0.72, 2.02	0.5	0.72	0.39, 1.35	0.3
11–20	48 (20%)	68 (23%)	0.99	0.59, 1.64	>0.9	0.88	0.49, 1.56	0.7
5–10	88 (37%)	88 (30%)	1.40	0.89, 2.21	0.15	1.09	0.65, 1.83	0.7
Type of livestock reared								
Goat/Sheep	23 (9.6%)	42 (14%)	*Ref*	–		–	–	
Cattle	135 (56%)	177 (61%)	1.39	0.81, 2.46	0.2	1.27	0.66, 2.50	0.5
Buffalo	7 (2.9%)	1 (0.3%)	12.8	2.10, 247	0.021	6.41	0.88, 132	0.11
Mixed	75 (31%)	70 (24%)	1.96	1.08, 3.62	0.029	1.55	0.79, 3.11	0.2
Herd/flock size								
<5	85 (35%)	157 (54%)	*Ref*	–		–	–	
>50	17 (7.1%)	5 (1.7%)	6.28	2.39, 19.6	<0.001	3.95	1.25, 14.3	0.025
21–50	16 (6.7%)	6 (2.1%)	4.93	1.95, 14.2	0.001	3.79	1.28, 12.4	0.020
5–20	122 (51%)	122 (42%)	1.85	1.29, 2.66	<0.001	1.85	1.20, 2.89	0.006
Have you ever received formal training on animal health or disease prevention?								
No	149 (62%)	252 (87%)	*Ref*	–		–	–	
Yes	91 (38%)	38 (13%)	4.05	2.66, 6.28	<0.001	2.78	1.70, 4.62	<0.001
Do you practice semi-intensive or free grazing management for your animals?								
No	108 (45%)	150 (52%)	*Ref*	–		–	–	
Yes	132 (55%)	140 (48%)	1.31	0.93, 1.85	0.12	1.20	0.79, 1.84	0.4

Abbreviations: AOR, adjusted odds ratio; CI, confidence interval; COR, crude odds ratio. Reference categories: Age: <30 years; Gender: Male; Educational level: No formal education; Place of residence: Rural; Farming experience: <5 years; Type of livestock reared: Goat/Sheep; Herd/flock size: <5 animals; Formal training on animal health or disease prevention: No; Semi-intensive or free grazing management: No. *P* < 0.05 was considered statistically significant.

### 3.6 Correlation among KAP components

Spearman's rank correlation analysis was performed to examine the relationships among knowledge, attitude, and practice (KAP) domains (Table 6; Fig. 5, Fig. 6). The analysis revealed a moderate and statistically significant positive correlation between knowledge and attitudes (ρ = 0.574, *p* < 0.001), indicating that respondents with higher knowledge levels tended to demonstrate more favorable attitudes toward tick and tick-borne disease prevention. A moderate positive correlation was also observed between knowledge and practices (ρ = 0.410, *p* < 0.001), while a weaker yet significant positive correlation was found between attitudes and practices (ρ = 0.364, *p* < 0.001).

**Table 6:** Spearman's rank correlation among respondents on knowledge, attitude & practice regarding Tick and Tick-borne disease.

Association	Correlation(ρ)	*P* value
Knowledge and Attitudes	0.574	<0.001
Knowledge and Practices	0.410	<0.001
Attitudes and Practices	0.364	<0.001

**Fig. 5:**
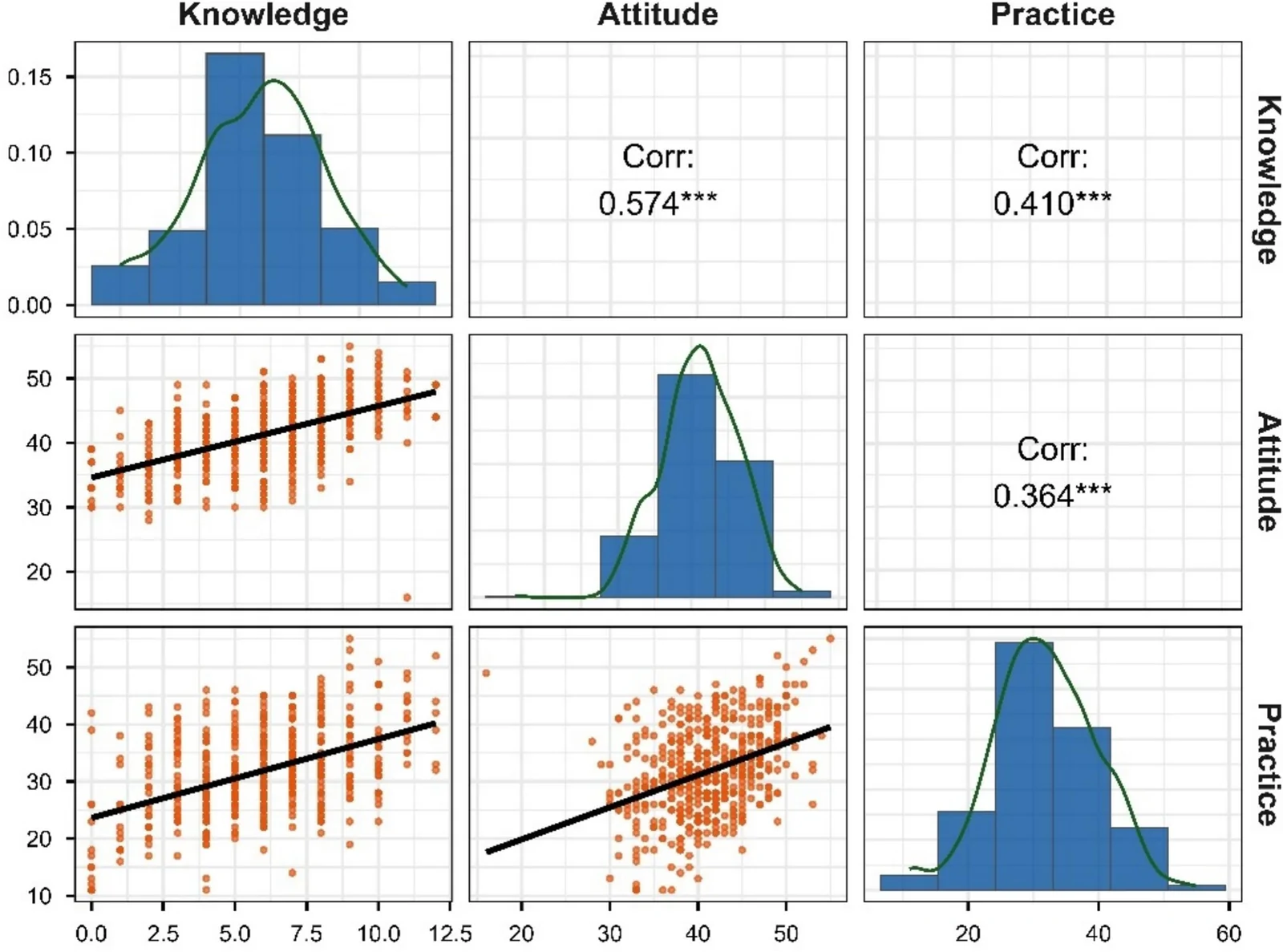
Scatter plot correlation (Spearman's correlation) matrix with histogram showing the correlation matrix among the knowledge, attitude, and practice level.

**Fig. 6:**
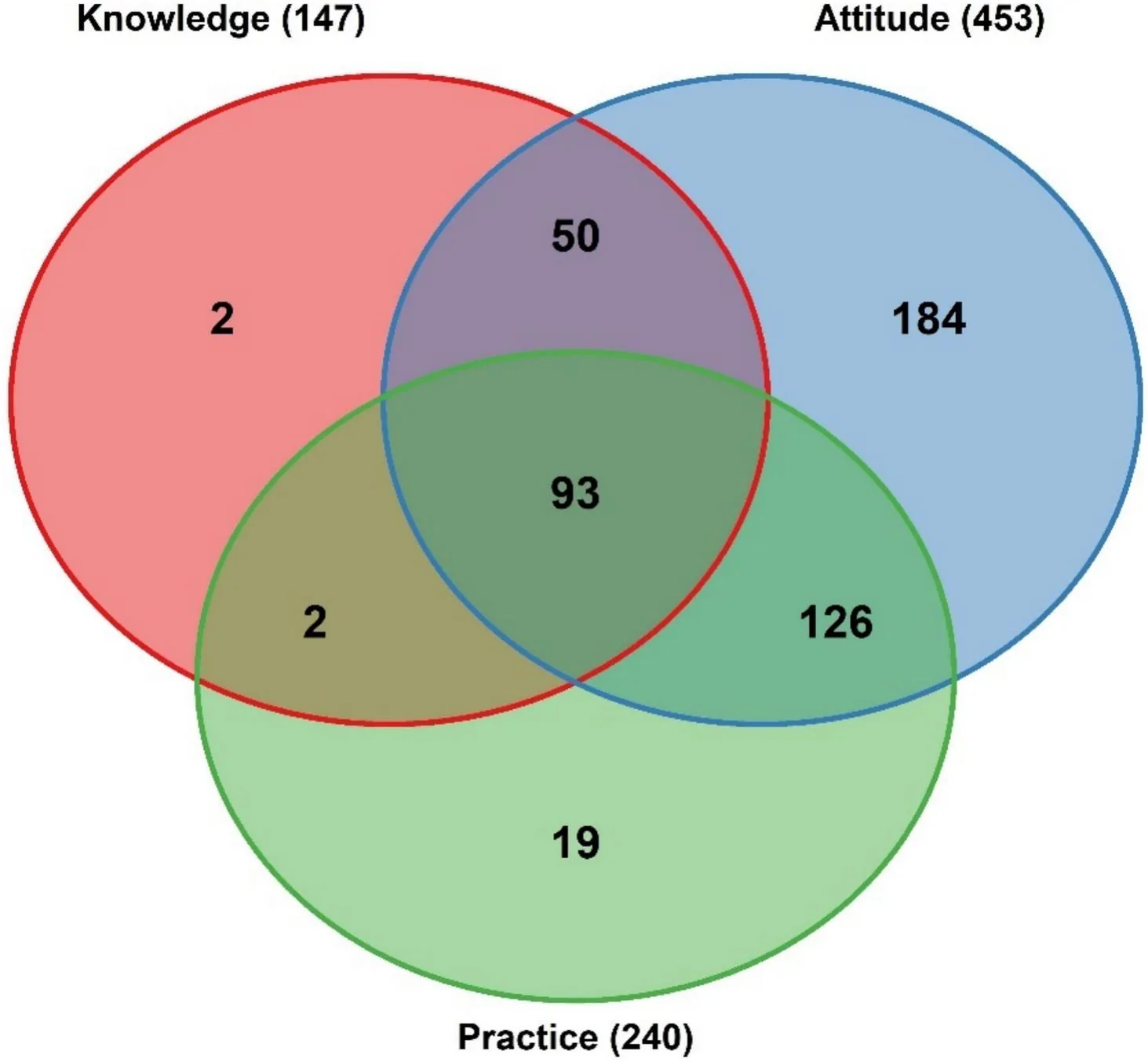
A Venn diagram showing the knowledge, attitude, and practice level among the participants.

## 4 Discussion

KAP studies are widely used in public health and veterinary epidemiology to evaluate community awareness, perceptions, and behavioral practices related to disease prevention and control. By examining the knowledge, attitudes, and practices of livestock farmers, researchers can gain useful insights into existing gaps in awareness and management. This information helps in developing targeted educational programs, improving veterinary extension services, and strengthening strategies aimed at preventing and controlling diseases (Yizengaw et al., 2025). This study explored the knowledge, attitudes, and practices (KAP) of livestock farmers regarding ticks and tick-borne diseases across several districts in Bangladesh. To the best of our knowledge, it is among the first studies to provide a comprehensive overview of these aspects among livestock farmers in the country. The results indicate that while farmers generally showed positive attitudes toward the control and prevention of TTBDs, their overall knowledge about these diseases was limited. In addition, preventive practices were often insufficient. These findings highlight important gaps in awareness and management of TTBDs among livestock farmers and emphasize the need for improved education and support in this area.

In the present study, only 27.7% of the respondents showed good knowledge about TTBDs, while the majority had a low level of understanding. Most farmers were able to recognize ticks as blood-feeding ectoparasites (78.5%), but awareness of other important aspects was much lower. For example, relatively few farmers knew about the possibility of zoonotic transmission (32.1%), acaricide resistance (26.8%), or specific tick-borne diseases such as anaplasmosis, babesiosis, and theileriosis (23.4%). This limited level of knowledge may be linked to insufficient veterinary extension services, a lack of farmer training programs, and the continued reliance on traditional livestock management practices in many rural areas of Bangladesh. Similar findings were reported in livestock-dependent communities, where 25% of farmers in Madhya Pradesh, India (Jamra et al., 2024) and 59% in South Africa lacked awareness of the impact of tick-borne diseases on cattle productivity (Yawa et al., 2020). In contrast, relatively higher knowledge was reported in Bhutan (52%) (Namgyal et al., 2021) and in a study by Chakraborty et al. (2023), where about 60% of respondents had at least moderate knowledge of ticks (Chakraborty et al., 2023). This limited level of knowledge could have important consequences in Bangladesh. Low awareness may increase the risk of tick infestations and the spread of tick-borne diseases, thereby reduce cattle productivity and cause economic losses for livestock farmers. In addition, the potential for zoonotic transmission means that these diseases may also pose risks to public health. The study found several factors that were linked to a better understanding of TTBDs among farmers. Women tended to have more knowledge than men, and graduates were more likely to be well-informed than farmers with less formal education. Farmers who had been working in the field for 5–20 years, as well as those managing herds of over 50 animals, also showed higher levels of knowledge. This could be because women often take on daily care of livestock, while higher education, longer experience, and larger herd sizes provide more opportunities to encounter ticks, learn about disease prevention, and interact with veterinarians.

In the present study, respondents' attitudes toward tick prevention and control were more positive than their knowledge levels, with a majority (85.5%) expressing favorable attitudes toward tick control and disease prevention. Most farmers recognized tick infestation as a serious livestock health problem (71.4%) and agreed that preventive measures are essential for farm management. Many acknowledged that prevention is more effective than treatment (62.5%) and that poor farm hygiene increases tick risk (77.5%), while fewer emphasized regular acaricide use (43.4%) and veterinary consultation (49.5%). The comparatively higher positive attitude observed in this study may be attributed to farmers' direct experience with tick infestation and its economic impact on livestock productivity, which likely increases their willingness to adopt preventive and control measures even in the presence of limited formal knowledge. Similarly, KAP studies among cattle farmers reported that 68.7% in Bhutan (Namgyal et al., 2021) and 51.7% in Africa (Khamassi Khbou et al., 2025) had positive attitudes toward tick and tick-borne disease prevention, respectively. Other studies reported lower favorable attitudes, with 47.86% in Malaysia (Khang et al., 2025) and 36.5% in India (Jamra et al., 2024).

The current research indicated that farmers with formal training in animal health tended to exhibit more favorable attitudes. Positive attitudes were generally elevated among individuals raising mixed livestock and residing in urban locations, although these relationships were not statistically significant. Their more favorable attitudes were likely attributed to increased exposure to animal health information and hands-on experience with tick management. Despite relatively positive attitudes, preventive practices among the respondents were relatively poor, with only 45.3% demonstrating satisfactory practices. Regular tick inspection of animals was not consistently performed, and only 15.5% of farmers reported always using acaricides for tick control. Similarly, adherence to recommended acaricide dosage and application instructions was low (13.2%), and veterinary consultation was infrequent (7.7%). Many respondents relied on traditional methods or irregular control measures rather than implementing systematic tick management strategies. Similarly, 81.3% of patients used protective measures against tick bites, and 68.3% reported infestations to a veterinarian, yet gaps in knowledge and proper reporting practices remained (Malgwi et al., 2025). In the Democratic Republic of Congo, 84.5% of farmers practiced spraying and 77.2% performed tick inspections (Mutwedu et al., 2025), while in Plateau State, Nigeria, 58.8% (Ogo et al., 2023) and in Sindh, Pakistan, 42.5% (Rajput et al., 2023) relied on manual tick removal with few protective measures. The variations probably result from differences in farmers' expertise, education, and availability of veterinary services, along with a cultural preference for traditional methods rather than organized tick control. This research indicated that farmers with formal training, higher education levels, larger herds, and greater age were more inclined to exhibit effective practices related to tick management and tick-borne illnesses. This is probably due to training, education, more extensive herd management, and age, offering increased knowledge, experience, and understanding of effective tick control methods. Environmental and management factors may also influence tick prevalence and exposure risk. Previous epidemiological studies have demonstrated that grazing practices, herd management systems, and housing conditions play significant roles in tick infestation rates. For example, livestock raised under grazing systems were reported to be six times more susceptible to heavy tick infestations compared to animals managed under stall-feeding systems (Rehman et al., 2017). These findings highlight the importance of livestock management practices in determining tick exposure and underline the need for improved husbandry and biosecurity measures.

The findings of this study provide important baseline information on livestock farmers' knowledge, attitudes, and practices regarding ticks and tick-borne diseases in Bangladesh. Although this study primarily focused on the animal health component by assessing farmers' awareness and preventive behaviors, the findings have implications for a broader One Health perspective, which recognizes the interconnectedness of human, animal, and environmental health (Eremeeva and Das, 2024). The identified gaps in knowledge, particularly regarding zoonotic transmission and appropriate tick control practices, may contribute to increased risks at the animal–human interface. Addressing these challenges requires improved farmer education, strengthened veterinary extension services, and sustainable tick management strategies (Makwarela et al., 2025). Future studies incorporating human health surveillance, environmental tick monitoring, pathogen detection, and collaboration among veterinary and medical sectors are warranted to develop comprehensive One Health strategies for tick-borne disease prevention in Bangladesh.

### 4.1 Strengths and limitations

This study represents one of the first comprehensive assessments of livestock farmers' knowledge, attitudes, and practices (KAP) regarding ticks and tick-borne diseases in Bangladesh. The findings provide important insights into farmers' awareness and preventive behaviors while highlighting critical gaps that may serve as baseline evidence for targeted tick control interventions and future One Health-oriented research. However, several limitations should be considered when interpreting the results. Data were obtained through self-reported questionnaires and may therefore be subject to recall bias or socially desirable responses. In addition, the cross-sectional design precludes causal inference between the variables examined. The knowledge score was derived from responses to a set of questions and, although somewhat simplified, may not fully capture the relative weight of each item. Furthermore, this study primarily focused on the animal health component and did not include human health assessments, environmental tick surveillance, or laboratory-based pathogen investigations. Nevertheless, it provides a reasonable approximation of participants' knowledge levels. Despite these limitations, the study offers valuable baseline evidence that may assist public health and veterinary authorities in Bangladesh in developing targeted awareness programs and evidence-based strategies for tick control and disease prevention. Further research integrating epidemiological, environmental, and laboratory approaches along with human health surveillance and intersectoral collaboration is warranted to better characterize the risks associated with ticks and tick-borne diseases and to support the development of comprehensive One Health strategies aimed at improving both animal and human health outcomes.

## 5 Conclusion

This study provides critical insights into livestock farmers' knowledge, attitudes, and practices regarding ticks and tick-borne diseases in Bangladesh, revealing a substantial gap between generally positive attitudes and inadequate knowledge and preventive practices. Although most farmers recognize the importance of tick control, limited awareness of zoonotic risks, acaricide resistance, and appropriate management strategies, along with poor adherence to recommended practices, may facilitate the persistence and spread of TTBDs. Education, farming experience, herd size, and formal training were identified as key determinants of improved knowledge and practices, while significant positive correlations among KAP domains suggest that enhancing knowledge can promote more favorable attitudes and better behavioral outcomes. These findings highlight the urgent need for targeted, context-specific interventions, including strengthened veterinary extension services, farmer education, and the promotion of integrated tick management strategies. Future implementation of a One Health approach integrating animal, human, and environmental health components will be essential for developing comprehensive surveillance and control strategies, reducing zoonotic risks, and improving livestock health and productivity in Bangladesh.

## CRediT authorship contribution statement

**Md. Hasan Ali:** Writing – review & editing, Writing – original draft, Visualization, Software, Methodology, Investigation, Formal analysis, Data curation, Conceptualization. **Hemayet Hossain:** Writing – review & editing, Writing – original draft, Visualization, Software, Methodology, Investigation. **Niaz Mahmood Tanoy:** Writing – original draft, Methodology, Investigation. **Md. Tanvir Hasan Bhuiyan:** Writing – original draft, Methodology, Data curation. **Nandiny Saha Roy:** Writing – original draft, Methodology, Investigation. **Md. Zubayer Islam:** Methodology, Investigation, Data curation. **Md. Saiful Islam:** Methodology, Investigation, Data curation. **Nitto Roy:** Methodology, Investigation, Data curation. **Sherin Akter Shimu:** Methodology, Investigation, Data curation. **Md. Ashraful:** Methodology, Investigation, Data curation. **Aliful Islam Shykat:** Methodology, Investigation, Data curation. **Athoy Das:** Methodology, Investigation, Data curation. **Md. Mahfujur Rahman:** Writing – review & editing, Writing – original draft, Visualization, Validation, Supervision, Software, Resources, Project administration, Methodology, Investigation, Formal analysis, Data curation, Conceptualization.

## Funding

No Funding.

## Declaration of competing interest

The authors declare that they have no known competing financial interests or personal relationships that could have appeared to influence the work reported in this paper.

## Data Availability

Data are available to the corresponding author upon reasonable request.

## Appendix A Supplementary data

**Supplementary materials** Supplementary Table 1: Distribution of participants’ knowledge regarding ticks and tick-borne diseases (N = 530). Supplementary Table 2: Distribution of participants’ attitudes regarding ticks and tick-borne diseases (N = 530). Supplementary Table 3: Distribution of participants’ practices regarding ticks and tick-borne diseases (N = 530).
