# Supplementary material for: Assessment of knowledge, attitudes, and practices among livestock farmers regarding ticks and tick-borne diseases in Bangladesh: A cross-sectional study
Source: Parasite Epidemiol Control. 2026 Sep 30;35:e00549. doi: 10.1016/j.parepi.2026.e00549 (PMC13659613; doi:10.1016/j.parepi.2026.e00549)
Supplement: Supplementary file 1 [file mmc1.docx]

| **Supplementary Table 1.**  Distribution of participants’ knowledge regarding ticks and tick-borne diseases (N = 530). | |
| --- | --- |
| **Characteristic** | **N = 530***^1^* |
| Are ticks external parasites that feed on the blood of animals? |  |
| Yes | 416 (78.5%) |
| No | 41 (7.7%) |
| Don’t know | 73 (13.8%) |
| Can ticks transmit infectious diseases to livestock animals? |  |
| Yes | 294 (55.5%) |
| No | 88 (16.6%) |
| Don’t know | 148 (27.9%) |
| Do tick-borne diseases reduce animal productivity, such as milk yield, meat production, or growth rate? |  |
| Yes | 345 (65.1%) |
| No | 49 (9.2%) |
| Don’t know | 136 (25.7%) |
| Can untreated tick-borne diseases cause severe illness or death in animals? |  |
| Yes | 225 (42.5%) |
| No | 145 (27.4%) |
| Don’t know | 160 (30.2%) |
| Can ticks transmit diseases from animals to humans (zoonotic transmission)? |  |
| Yes | 170 (32.1%) |
| No | 162 (30.6%) |
| Don’t know | 198 (37.4%) |
| Does warm and humid weather favor the survival and reproduction of ticks? |  |
| Yes | 211 (39.8%) |
| No | 93 (17.5%) |
| Don’t know | 226 (42.6%) |
| Is tick infestation more common during specific seasons of the year? |  |
| Yes | 219 (41.3%) |
| No | 131 (24.7%) |
| Don’t know | 180 (34.0%) |
| Are free-grazing or pasture-raised animals more exposed to tick infestation than stall-fed animals? |  |
| Yes | 259 (48.9%) |
| No | 95 (17.9%) |
| Don’t know | 176 (33.2%) |
| Can proper tick control measures effectively prevent tick-borne diseases? |  |
| Yes | 361 (68.1%) |
| No | 46 (8.7%) |
| Don’t know | 123 (23.2%) |
| Can incorrect or excessive use of acaricides (chemicals used to kill or control ticks) lead to resistance in ticks? |  |
| Yes | 142 (26.8%) |
| No | 118 (22.3%) |
| Don’t know | 270 (50.9%) |
| Does early diagnosis and timely treatment improve recovery from tick-borne diseases in animals? |  |
| Yes | 349 (65.8%) |
| No | 43 (8.1%) |
| Don’t know | 138 (26.0%) |
| Do you know that ticks transmit diseases such as anaplasmosis, babesiosis, theileriosis, and other blood-borne infections in livestock and humans? |  |
| Yes | 124 (23.4%) |
| No | 140 (26.4%) |
| Don’t know | 266 (50.2%) |

| **Supplementary Table 2.**  Distribution of participants’ attitudes regarding ticks and tick-borne diseases (N = 530). | | |
| --- | --- | --- |
| **Characteristic** | **N = 530***^1^* | |
| Tick infestation is a serious problem affecting livestock health. |  | |
| Strongly agree | 136 (25.7%) | |
| Agree | 242 (45.7%) | |
| Neutral | 121 (22.8%) | |
| Disagree | 27 (5.1%) | |
| Strongly disagree | 4 (0.8%) | |
| Controlling ticks should be a regular and essential part of farm management. |  | |
| Strongly agree | 115 (21.7%) | |
| Disagree | 23 (4.3%) | |
| Agree | 220 (41.5%) | |
| Neutral | 167 (31.5%) | |
| Strongly disagree | 5 (0.9%) | |
| Tick-borne diseases cause significant economic losses to livestock farmers. |  | |
| Strongly agree | 96 (18.1%) | |
| Agree | 231 (43.6%) | |
| Neutral | 162 (30.6%) | |
| Disagree | 38 (7.2%) | |
| Strongly disagree | 3 (0.6%) | |
| Preventing tick infestation is more effective than treating tick-borne diseases. |  | |
| Strongly agree | 119 (22.5%) | |
| Agree | 212 (40.0%) | |
| Neutral | 168 (31.7%) | |
| Disagree | 29 (5.5%) | |
| Strongly disagree | 2 (0.4%) | |
| Farmers play an important role in controlling tick infestation on their farms. |  | |
| Strongly agree | 153 (28.9%) | |
| Agree | 217 (40.9%) | |
| Neutral | 142 (26.8%) | |
| Disagree | 17 (3.2%) | |
| Strongly disagree | 1 (0.2%) | |
| Regular use of acaricides (chemicals used to kill or control ticks) is necessary for effective tick control. |  | |
| Strongly agree | 64 (12.1%) | |
| Agree | 166 (31.3%) | |
| Neutral | 209 (39.4%) | |
| Disagree | 81 (15.3%) | |
| Strongly disagree | 10 (1.9%) | |
| Veterinary consultation is important for the proper management of tick-borne diseases. |  | |
| Strongly agree | 81 (15.3%) | |
| Agree | 181 (34.2%) | |
| Neutral | 188 (35.5%) | |
| Disagree | 74 (14.0%) | |
| Strongly disagree | 6 (1.1%) | |
| Poor farm hygiene increases the risk of tick infestation. |  | |
| Strongly agree | 272 (51.3%) | |
| Agree | 139 (26.2%) | |
| Neutral | 93 (17.5%) | |
| Disagree | 23 (4.3%) | |
| Strongly disagree | 3 (0.6%) | |
| Reporting severe tick infestation or disease outbreaks to veterinarians is important. |  | |
| Strongly agree | 58 (10.9%) | |
| Agree | 209 (39.4%) | |
| Neutral | 210 (39.6%) | |
| Disagree | 49 (9.2%) | |
| Strongly disagree | 4 (0.8%) | |
| Training farmers on tick control practices would reduce the occurrence of tick-borne diseases. |  | |
| Strongly agree | 72 (13.6%) | |
| Agree | 253 (47.7%) | |
| Neutral | 177 (33.4%) | |
| Disagree | 24 (4.5%) | |
| Strongly disagree | 4 (0.8%) | |
| Training farmers on tick control practices would reduce the occurrence of tick-borne diseases. |  | |
| Strongly agree | 78 (14.7%) | |
| Disagree | 22 (4.2%) | |
| Agree | 251 (47.4%) | |
| Neutral | 177 (33.4%) | |
| Strongly disagree | 2 (0.4%) | |
| **Supplementary Table 3.**  Distribution of participants’ practices regarding ticks and tick-borne diseases (N = 530). | | |
| **Characteristic** | | **N = 530***^1^* |
| I regularly check my animals for ticks. | |  |
| Always | | 161 (30.4%) |
| Never | | 24 (4.5%) |
| Often | | 136 (25.7%) |
| Rarely | | 57 (10.8%) |
| Sometimes | | 152 (28.7%) |
| I remove visible ticks from animals manually or mechanically. | |  |
| Always | | 218 (41.1%) |
| Never | | 31 (5.8%) |
| Often | | 98 (18.5%) |
| Rarely | | 89 (16.8%) |
| Sometimes | | 94 (17.7%) |
| I use acaricides (chemicals used to kill or control ticks) to control tick infestation. | |  |
| Always | | 82 (15.5%) |
| Never | | 129 (24.3%) |
| Often | | 62 (11.7%) |
| Rarely | | 101 (19.1%) |
| Sometimes | | 156 (29.4%) |
| I follow veterinary or manufacturer instructions for dosage and application of acaricides. | |  |
| Always | | 70 (13.2%) |
| Never | | 138 (26.0%) |
| Often | | 84 (15.8%) |
| Rarely | | 119 (22.5%) |
| Sometimes | | 119 (22.5%) |
| I clean and maintain animal housing to reduce tick habitats. | |  |
| Always | | 257 (48.5%) |
| Never | | 22 (4.2%) |
| Often | | 81 (15.3%) |
| Rarely | | 72 (13.6%) |
| Sometimes | | 98 (18.5%) |
| I seek veterinary advice when animals show signs of tick-borne diseases. | |  |
| Always | | 41 (7.7%) |
| Never | | 101 (19.1%) |
| Often | | 76 (14.3%) |
| Rarely | | 158 (29.8%) |
| Sometimes | | 154 (29.1%) |
| I isolate heavily infested or sick animals from healthy ones. | |  |
| Always | | 61 (11.5%) |
| Never | | 122 (23.0%) |
| Often | | 86 (16.2%) |
| Rarely | | 136 (25.7%) |
| Sometimes | | 125 (23.6%) |
| I treat newly purchased animals for ticks before mixing them with the herd. | |  |
| Always | | 79 (14.9%) |
| Never | | 122 (23.0%) |
| Often | | 71 (13.4%) |
| Rarely | | 121 (22.8%) |
| Sometimes | | 137 (25.8%) |
| I rotate or manage grazing areas to minimize tick exposure. | |  |
| Always | | 55 (10.4%) |
| Never | | 212 (40.0%) |
| Often | | 41 (7.7%) |
| Rarely | | 108 (20.4%) |
| Sometimes | | 114 (21.5%) |
| I keep records of tick infestation, disease occurrence, and treatments administered. | |  |
| Always | | 51 (9.6%) |
| Never | | 233 (44.0%) |
| Often | | 65 (12.3%) |
| Rarely | | 86 (16.2%) |
| Sometimes | | 95 (17.9%) |
| I use personal protective measures (e.g., gloves or protective clothing) when handling tick-infested animals. | |  |
| Always | | 30 (5.7%) |
| Never | | 154 (29.1%) |
| Often | | 77 (14.5%) |
| Rarely | | 127 (24.0%) |
| Sometimes | | 142 (26.8%) |
